# Using Acupuncture for Adjunct Treatment for Cancer-Related Fatigue in Breast Cancer Patients Is Practicable. Reply to Hu et al. Is Acupuncture an Ideal Adjunctive Treatment for Cancer-Related Fatigue? Comment on “Choi et al. Acupuncture for Managing Cancer-Related Fatigue in Breast Cancer Patients: A Systematic Review and Meta-Analysis. *Cancers* 2022, *14*, 4419”

**DOI:** 10.3390/cancers15082227

**Published:** 2023-04-10

**Authors:** Terje Alraek, Stephen Birch, Tae-Young Choi, Lin Ang, Ji Hee Jun, Weidong Lu, Myeong Soo Lee

**Affiliations:** 1 School of Health Sciences, Kristiania University College, 0107 Oslo, Norway; 2 The National Research Center in Complementary and Alternative Medicine (NAFKAM), Department of Community Medicine, Faculty of Health Science, UiT, The Arctic University of Norway, 9037 Tromsø, Norway; 3 KM Science Research Division, Korea Institute of Oriental Medicine, Daejeon 34054, Republic of Korea; 4 Dana-Farber Cancer Institute, Harvard Medical School USA, Boston, MA 02215, USA

We are grateful for the relevant comments by Hu et al. [1] for our systematic review [2]. We would like to respond to their comments. First, the authors have described the “lack of objectivity and universality of the conclusion of the meta-analysis conclusion”, but perhaps they misunderstand the nature of the systematic review. We assessed the clinical evidence of acupuncture based on a systematic approach; the nature of our study is objective, and the lack of generality is obvious, as we only included randomized controlled trials. The authors also called our study a meta-analysis only (it is a statistical approach as part of a systematic review), but our study is a systematic review. Our conclusion is based on the analysis of the included studies and not only on the results of the meta-analysis. Second, the authors raised issues with searching for recent studies and recommend searching a wider range of databases. We searched PubMed instead of Medline. PubMed is a larger database as it includes citations from Medline. The recommendation to search AMED seems reasonable, but from our experience, searches here seldom add new information as its scope of coverage is limited. Nevertheless, we searched AMED as recommended, but it did not provide us with any new findings, as expected. PsycINFO is generally used for psychological research and is not directly related to our research question. Therefore, Hu et al.’s arguments about more recent studies and searches in other databases seems weak. We believed that we performed our search comprehensively and reported an extensive search strategy. Third, the authors recommend using network meta-analysis (NMA) to find comparative effects with other interventions. This method may be useful depending on the research question, but it is not appropriate for our research question. One of the main problems with acupuncture NMA includes the use of placebo as a common control. The use of sham acupuncture as a placebo control is controversial. The use of placebo exercises, sham acupuncture, and several types of placebo control as common nodes seems questionable, and cannot provide us with an exact answer. Fourth, the authors recommend subgroup analysis. It seems that the authors did not read our paper carefully, especially Table 1. Most studies recruited patients at different stages of treatment, which is a reality in cancer studies or other clinical trials. We did not ignore the possibility of subgroup analysis but were unable to perform this.

In addition, we would like to address their thoughts on how to conduct a “[…] study to establish applicable guidelines and encourage standardized clinical practice”. As the authors write [1], the US NCCN recommends the use of acupuncture for cancer-related fatigue (CRF). Other countries have similar recommendations for acupuncture and CRF, e.g., Germany and Australia [3,4]. We also know that recognized US hospitals such as Memorial Sloan Kettering Cancer Center, Dana Farber and MD Anderson Cancer Center are offering acupuncture treatment based on the available evidence for various late effects, including CRF, and especially for cancer survivors. A recent publication [5] describes the National Ministry of Health, Department of Health, State Department of Health or National Health Service and national guideline groups recommending the use of acupuncture for cancer symptoms that are either from the cancer itself or from late effects such as fatigue. There are clear published recommendations on the safety of acupuncture and when to avoid it in cancer patients [6]. Likewise, there are a few clinical guidelines that do not recommend the use of acupuncture for certain symptoms in cancer patients.

It is common in medical practice to promote interventions based on evidence of their effectiveness and safety. At present, there are few effective therapies for CRF in breast cancer patients. Even though the evidence for the effectiveness of acupuncture is weak, this is also true for most other options. “Although evidence of the beneficial effects on fatigue outcomes is not particularly strong for interventions such as acupuncture, massage, or bright light, use of these therapies in clinical practice for fatigue management can be rationalized based on the fact that they are generally well tolerated and may be efficacious in particular fatigue contexts or may favorably affect symptoms that amplify fatigue, including anxiety, depression, sleep disturbance, and pain.” [7]. As Hu and colleagues state, it would also be good to study the additional effects of acupuncture in combination with interventions such as exercise. We also agree that “further investigation is warranted to validate whether AT yields similar effects for cancer patients in early and advanced disease trajectory”.
